# Automated Annuloplasty with VirtuoSEW^®^ in microInvasive Mitral Valve Repair (μMVr)

**DOI:** 10.3390/medsci14020187

**Published:** 2026-04-09

**Authors:** Nermir Granov, Farhad Bakhtiary, Armin Šljivo, Jude S. Sauer

**Affiliations:** 1 Clinic for Cardiovascular Surgery, Clinical Center of University of Sarajevo, Bolnička 25, 71000 Sarajevo, Bosnia and Herzegovina; 2 Department of Cardiac Surgery, University Hospital Bonn, 53127 Bonn, Germany; 3 LSI Solutions, Victor, NY 14564, USA; jsauer@lsisolutions.com

**Keywords:** mitral valve repair, automated annuloplasty, VirtuoSEW, mitral regurgitation, minimally invasive cardiac surgery

## Abstract

**Background/Objectives**: Totally endoscopic mitral valve repair reduces surgical trauma and accelerates recovery but can be technically challenging, particularly for precise annuloplasty suturing. The VirtuoSEW^®^ (LSI Solutions, Victor, NY 14564m, USA) automated annular suturing system was developed to standardize and simplify suture placement. This study was an early evaluation of this technology’s safety, efficacy, and feasibility in totally endoscopic microInvasive mitral valve repair (µMVr). **Methods**: We conducted a retrospective observational study of 20 patients with severe mitral valve disease of various etiologies. All patients underwent mitral valve repair using the VirtuoSEW^®^ system for automated placement of annuloplasty sutures, combined with leaflet resection or chordal management as appropriate. Postoperative outcomes were assessed at one month using echocardiography and clinical evaluation. Perioperative and postoperative complications and early mortality were systematically recorded. **Results**: VirtuoSEW^®^-assisted mitral valve repair was safe and effective, achieving complete elimination of severe mitral regurgitation in all patients (N = 20, 100%). Annuloplasty rings included Physio-ring (N = 12, 60%), Memo 3D (N = 4, 20%), and Memo 4D (N = 4, 20%), combined with leaflet repair techniques: leaflet plication (N = 5, 25%), neochordae implantation (N = 7, 35%), sliding plasty (N = 2, 10%), commissural repair (N = 1, 5%), and hemibutterfly repair (N = 1, 5%). Concomitant procedures included: tricuspid valve repair (N = 1, 5%) and atrial septal defect closure (N = 1, 5%). Mitral annulus diameter decreased from 42.0 ± 5.3 mm to 34.2 ± 2.2 mm (*p* = 0.001). Mean total surgery, cardiopulmonary bypass, and aortic cross-clamp times were 170.3 ± 21.3, 143.4 ± 21.5, and 80.4 ± 7.9 min, respectively. ICU stay was 1.0 ± 0.2 days, with a hospital stay of 8.0 ± 1.9 days. No perioperative complications—including bleeding (N = 0, 0%), stroke (N = 0, 0%), infections (N = 0, 0%), or 30-day mortality (N = 0, 0%)—occurred. **Conclusions**: µMVR invasive mitral valve repair using the VirtuoSEW^®^ system is safe, effective, and reproducible, as well as compatible with almost all repair techniques, providing complete restoration of valve competence with no early device-related complications. To our knowledge, this is the first clinical study reporting outcomes with this device, supporting its potential to streamline mitral repair and improve procedural efficiency.

## 1. Introduction

Totally endoscopic mitral valve repair (TEMVR) has gained popularity as a safe alternative to full sternotomy. Multiple studies and meta-analyses have shown that TEMVR offers comparable mortality and stroke rates to conventional sternotomy while providing significant clinical advantages, including shorter ICU and hospital stays, reduced need for blood transfusions, less postoperative pain, and faster overall recovery, establishing TEMVR as a safe and effective option in centers with sufficient experience [1,2,3,4]. A novel microInvasive mitral valve repair (µMVr) approach incorporates the use of endoscopic viewing with small intercostal access incisions to deliver the benefits of TEMVR while avoiding bone or cartilage injury.

Endoscopic access with inherently smaller working space restricts surgical maneuverability, which poses significant challenges for the accurate placement of annular sutures within the limited operative field. To overcome these technical limitations, the VirtuoSEW^®^ device has been developed [5]. The VirtuoSEW^®^ device is a long-shafted suturing instrument designed to simplify and standardize suture placement in minimally invasive procedures. With a single squeeze of the lever, the device deploys a curved needle to place half of a mattress suture, enabling consistent bite width and depth during remote suture delivery through limited access sites. It features an articulating jaw and rotational controls to optimize tip orientation and access to the suturing site. The needle remains safely retracted within the shaft until actuation, eliminating exposed sharps during handling. The device is compatible with various COR-SUTURE^®^ QUICK LOAD^®^ surgical sutures, allowing precise approximation of soft tissue and prosthetic materials [5]. This articulated automated annular suturing device facilitates the use of smaller, 15–25 mm surgical access sites, enabling µMVr.

Traditional hand-sewn annular suturing is associated with well-documented complications when technical execution is suboptimal [6,7,8]. Although infrequent, annular dehiscence can result in prosthetic ring detachment and acute mitral regurgitation. Other potential complications include inadvertent needle-related injuries, such as myocardial laceration, perforation of the atrial wall, injury to the atrioventricular conduction tissue, or damage to the circumflex coronary artery, which lies close to the mitral annulus. These risks underscore the necessity for precise needle control—a technical challenge that may be mitigated by automated annular suturing systems through standardized needle trajectories and reproducible suture placement [6,7].

The VirtuoSEW^®^ system offers several key benefits in the context of minimally invasive mitral valve repair [5]. By standardizing needle trajectory and stitch placement, the device enhances reproducibility and ergonomics within the constrained endoscopic operative field, ensuring uniform suture spacing and depth independent of individual surgeon experience. Manual annular suturing during minimally invasive procedures is technically challenging, requiring that long instruments are manipulated and needles are guided through narrow ports. Maintaining surgical precision through small, remote access is physically demanding and susceptible to human error [6,7]. Additionally, the VirtuoSEW^®^ minimizes exposure to free needle tips, thereby reducing the risk of glove punctures or inadvertent injury during suture handling [3].

The purpose of this study is to report the first experience in Bosnia and Herzegovina with the VirtuoSEW^®^ in μMVR. This study assessed the feasibility, safety, and effectiveness of the device in addressing the technical challenges of endoscopic surgery, including limited operative space and restricted maneuverability, while providing consistent and reproducible suture placement.

## 2. Materials and Methods

### 2.1. Study Design and Patient Population

This retrospective cross-sectional study was conducted between January 2023 and December 2025 at the Clinic for Cardiovascular Surgery, Clinical Center of the University of Sarajevo, Bosnia and Herzegovina. This study included 20 consecutive patients who underwent μMVR with the use of the VirtuoSEW^®^ (LSI Solutions, Victor, NY 14564m, USA) [5]. Patients with contraindications to minimally invasive mitral valve surgery, such as chest anomaly or previous chest operations, significant mitral annular calcification, distance of MV annulus/chest wall more than 25 cm and extensive pulmonary adhesion, were excluded from this study. Demographic, clinical, and procedural data were systematically collected and recorded in a dedicated institutional database.

All participants were thoroughly informed regarding the study objectives, procedural details, and potential outcomes, and written informed consent was obtained prior to inclusion. The study protocol received prior approval from the Institutional Ethics Committee of the Clinical Center of the University of Sarajevo **[177/23]** and was conducted in strict accordance with the Helsinki Declaration and its subsequent amendments.

### 2.2. Preoperative Assessment

Each patient underwent a thorough preoperative evaluation, which included a detailed medical history, assessment of comorbidities, coronary artery disease symptoms, medication and allergy history, electrocardiography, transthoracic echocardiography, CT scan or MRI and coronary angiography. Surgical risk was estimated using the Society of Thoracic Surgery (STS) risk score, incorporating patient demographics, laboratory values, comorbidities, and clinical characteristics to calculate the predicted 30-day mortality and morbidity [9].

### 2.3. Surgical Procedure

#### 2.3.1. Anesthesia and Positioning

All operations were performed under general anesthesia with endotracheal intubation. Patients were positioned supine with approximately 20° right hemithorax inflatable cushion elevation. Transesophageal echocardiography (TEE) was employed to guide cannulation and monitor cardiac and valve function throughout the procedure.

#### 2.3.2. Cannulation and Cardiopulmonary Bypass

CPB was established via right groin to reach the femoral artery and vein using the Seldinger technique under TEE guidance. Venous cannulation was achieved with 22–25 F multi-stage femoral cannulae, and arterial inflow was provided using 18–20 F femoral cannulae according to the patient’s body surface area.

#### 2.3.3. Right Micro-Thoracotomy Access (MICRO NEEDS TO STAY)

**Figure 1:**
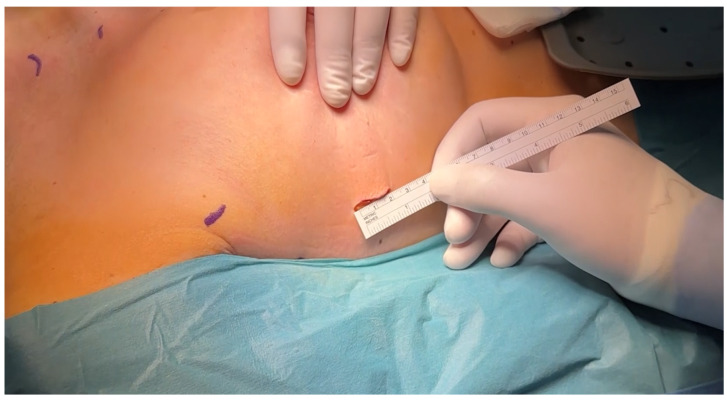
*Surgical approach for μMVR*.

**Figure 2:**
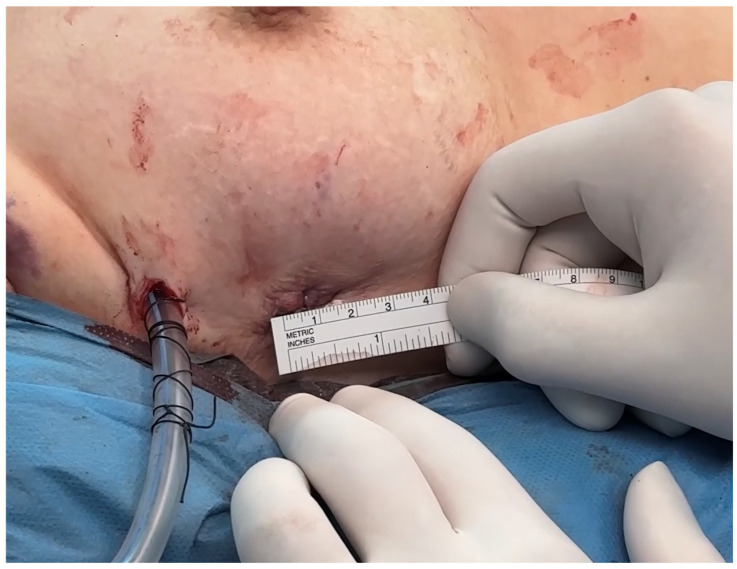
*The μMVR site sealed with liquid skin adhesive*.

A 15–25 mm incision was made at the third or fourth right intercostal space, in midaxillary line (Figure 1 and Figure 2). Soft tissue retractors were used to optimize exposure while preserving internal thoracic vessels. Two additional 10 mm ports were introduced to accommodate the 3D endoscopic camera mounted on multiSTATION^®^ (LSI Solutions, Victor, NY 14564m, USA) system, vent line and Chitwood aortic clamp. For pericardial retraction RAPORT™ Device Kit was used, and to secure cardioplegia line miniRUMEL^®^ (LSI Solutions, Victor, NY 14564m, USA) was used. Carbon dioxide insufflation was administered at 1.5–2 L/min to reduce the risk of air embolism.

### 2.4. VirtuoSEW^®^ Device Use in Valve Surgery

**Figure 3:**
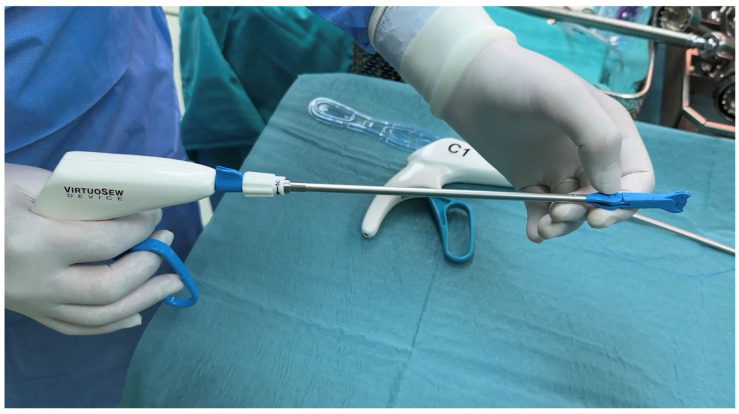
*VirtuoSEW™ (LSI Solutions, Victor, NY 14564m, USA) device*.

After the mitral valve was exposed through the left atrium via Waterston’s groove, anterior and posterior annular sutures were placed using the VirtuoSEW^®^ (LSI Solutions, Victor, NY 14564m, USA) (Figure 3).

**Figure 4:**
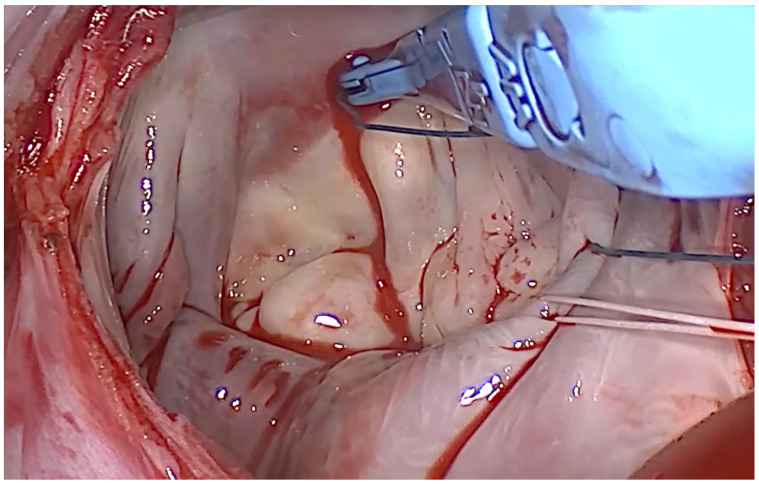
*Figure 4 at the points in Video 1*.

With a single lever squeeze, the VirtuoSEW^®^ (LSI Solutions, Victor, NY 14564m, USA) automated suturing device was used to deploy half of a horizontal mattress suture, with a consistent bite width of about 8–10 mm and depth of about 2–3 mm to the targeted annular tissue. The device’s articulating and rotational controls optimize tip orientation, and its design safeguards against exposed sharps by keeping the needle retracted within the shaft until actuation [5] (**Figure 4**).

**Figure 5:**
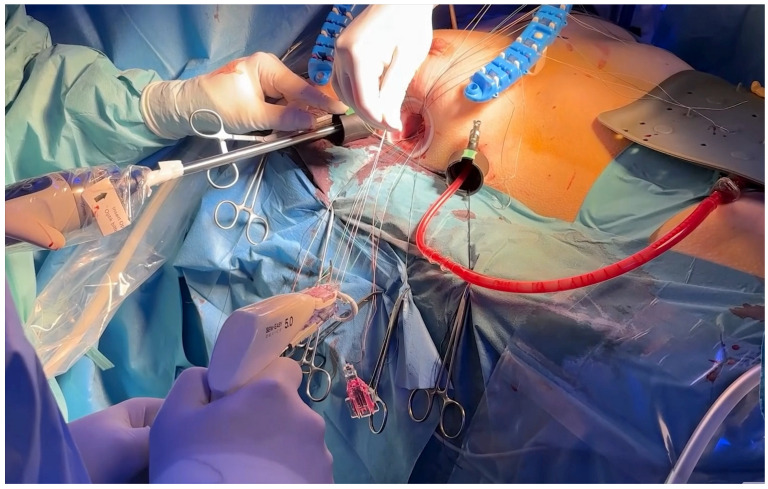
*Figure 5 at the points in Video 2*.

After each suture was placed through mitral annular tissue, both ends of the suture were transferred to SEW-EASY^®^ 5.0 (LSI Solutions, Victor, NY 14564m, USA) cassettes and placed in the RAM^®^ RING/RACK (LSI Solutions, Victor, NY 14564m, USA) organizer mounted on a multiSTATION^®^ (LSI Solutions, Victor, NY 14564m, USA). When annular suturing was complete, the SEW-EASY^®^ (LSI Solutions, Victor, NY 14564m, USA) device passed the ends of the annular suture through the annuloplasty prosthesis. The SEW-EASY^®^ (LSI Solutions, Victor, NY 14564m, USA) is compatible with a variety of different types and sizes of annuloplasty rings and bands (**Figure 5**).

After seating the annuloplasty ring, sutures were fastened with the COR-KNOT^®^ device (LSI Solutions, Victor, NY 14564m, USA), and a water leak test was performed. Additional mitral valve repair techniques (e.g., leaflet plication, neochordae implantation, sliding plasty) were subsequently carried out. The left atrium was closed in one layer of continuous 4–0 Prolene sutures. De-airing was achieved via the aortic vent catheter under TEE guidance.

### 2.5. Postoperative Data Collection and Follow-Up

Evaluated surgical metrics included: aortic cross-clamp and CPB times, total operative duration, ICU and hospital length of stay, transfusion requirements, PVL, inotropic support, wound healing, procedure-related complications, morbidity and mortality, myocardial infarction, stroke, delirium, acute kidney injury, new-onset atrial fibrillation, temporary pacemaker requirement, low cardiac output syndrome, wound revision, and respiratory failure requiring prolonged intubation or tracheostomy. All patients were followed for one month, with evaluations at 7 days and 30 days post-surgery, including laboratory tests, echocardiography, wound assessment, and clinical examination.

### 2.6. Statistical Analysis

All data were analyzed using IBM SPSS Statistics (version 28.0, IBM Corp., Armonk, NY, USA). Continuous variables were assessed for normality using the Kolmogorov–Smirnov test and visual inspection of Q–Q plots. Variables with a normal distribution are presented as mean ± standard deviation, while non-normally distributed variables are presented as median (interquartile range). Categorical variables are reported as frequencies and percentages. Comparative analyses between subgroups were performed using the independent samples *t*-test for normally distributed continuous variables or the Mann–Whitney U test for non-normally distributed variables, and the chi-square or Fisher’s exact test was performed for categorical variables, as appropriate. A *p*-value < 0.05 was considered statistically significant.

## 3. Results

A total of 20 patients were included in this study. The primary indication for surgery was severe mitral valve regurgitation, most commonly due to mitral valve prolapse N = 10 (50.0%), followed by degenerative mitral valve disease N = 5 (25.0%), ischemic severe mitral valve regurgitation N = 3 (15.0%), and Morbus Barlow N = 2 (10.0%). The study population consisted of 11 female (55.0%) and 9 male patients (45.0%). The median age was 57.5 years (interquartile range [IQR] 42.25–62.5). Median body weight was 90.0 kg (IQR 74.5–96.6), median height was 180.0 cm (IQR 172.5–185.2), and median body mass index was 27.3 kg/m^2^ (IQR 25.7–27.9). The most prevalent comorbidity was arterial hypertension (N = 10, 50.0%), followed by smoking (N = 5, 25.0%), hyperlipidemia (N = 3, 15.0%), diabetes mellitus (N = 2, 10.0%), chronic kidney disease (N = 2, 10.0%), peripheral artery disease (N = 2, 10.0%), and chronic obstructive pulmonary disease (N = 1, 5.0%). The mean STS operative mortality was 0.82% (0.39%; 1.23%) (range 0.24–1.77%), while the mean STS morbidity and mortality was 6.81% (5.25%; 8.72%) (range 4.89–8.96%). Other baseline characteristics are summarized in Table 1.

Coronary angiography demonstrated that the majority of patients had no significant coronary artery disease. Three patients were diagnosed with triple-vessel disease of not significant stenosis. Two patients had significant coronary artery stenoses, both involving the right coronary artery, which were successfully treated with drug-eluting stents more than one year prior to surgery. No cases of perioperative myocardial infarction were observed in the study cohort.

Following mitral valve repair performed with the VirtuoSEW^®^ automated annular suturing system, clinically meaningful improvement was observed. Severe mitral regurgitation, present in all patients preoperatively (N = 20, 100%), was completely eliminated postoperatively and remained absent at follow-up (0%).

The minimum follow-up period was 6 months postoperatively, with a mean follow-up duration of 9.4 ± 2.1 months. It should be noted that follow-up scheduling was not entirely uniform due to administrative and bureaucratic differences between cantons, which influenced the timing of outpatient echocardiographic reassessment.

Echocardiographic assessment at about 1 month postoperatively demonstrated a significant reduction in mitral valve annular diameter, decreasing from 42.0 ± 5.3 mm preoperatively to 34.2 ± 2.2 mm postoperatively and remaining stable at follow-up (34.2 ± 2.1 mm, *p* = 0.001). In parallel, vena contracta width was markedly reduced from 8.4 ± 1.3 mm to 2.0 ± 1.6 mm postoperatively (*p* < 0.001), with sustained results at follow-up. Regurgitant volume significantly decreased from 73.5 ± 9.3 mL preoperatively to 5.5 ± 5.3 mL postoperatively (*p* = 0.019), confirming durable elimination of significant mitral regurgitation.

Left ventricular systolic function remained preserved throughout the observation period. Ejection fraction showed no statistically significant change (52.5 ± 10.9% preoperatively vs. 51.5 ± 11.2% postoperatively vs. 53.0 ± 8.1% at follow-up; *p* = 0.471). However, significant reverse remodeling was observed in left ventricular dimensions, with LVIDd decreasing from 58.1 ± 5.9 mm to 52.7 ± 5.9 mm postoperatively and 52.0 ± 2.4 mm at follow-up (*p* < 0.001), and LVIDs decreasing from 37.2 ± 9.0 mm to 35.7 ± 6.9 mm postoperatively and 35.7 ± 10.9 mm at follow-up (*p* < 0.001).

Left atrial diameter was also significantly reduced from 58.0 ± 9.6 mm preoperatively to 52.1 ± 7.2 mm postoperatively and 52.7 ± 7.5 mm at follow-up (*p* = 0.019). Interventricular septal thickness and posterior wall thickness remained stable (*p* = 0.230 for both). Right ventricular systolic function assessed by TAPSE showed no significant differences across time points (23.6 ± 3.8 mm, 23.8 ± 5.2 mm, and 24.2 ± 4.3 mm, *p* = 0.275). Right ventricular systolic pressure also remained stable, decreasing numerically from 37.0 (29.0–56.7) mmHg preoperatively to 36.5 (25.7–53.7) mmHg postoperatively and 30.1 (24.2–45.6) mmHg at follow-up (*p* = 0.109).

Aortic valve parameters, including diameter (26.1 ± 3.1 mm), velocity (1.4 ± 0.2 m/s), and mean pressure gradient (7.4–7.5 ± ~3.6 mmHg), remained unchanged during follow-up. Severe tricuspid regurgitation was present in 3 patients (15.0%) preoperatively, persisted in 2 patients (15.0%) postoperatively, and was absent at follow-up.

Overall, mitral valve repair using the VirtuoSEW^®^ system resulted in complete elimination of severe mitral regurgitation, significant annular reduction, reverse cardiac remodeling, and preserved biventricular systolic function with stable hemodynamic parameters at follow-up. All remaining echocardiographic parameters and statistical comparisons are presented in detail in Table 2.

Mitral valve repair was performed in all 20 patients (N = 20, 100%), using annuloplasty rings—Physio-ring in N = 12 (60%), Memo 3D in N = 4 (20%), and Memo 4D in N = 4 (20%)—combined with various leaflet repair techniques, including leaflet plication (N = 5, 25%), neochordae implantation (N = 7, 35%), sliding plasty (N = 2, 10%), commissural repair (N = 1, 5%), and hemibutterfly repair (N = 1, 5%). Concomitant procedures were uncommon, with tricuspid valve repair (N = 1, 5%) and atrial septal defect closure (N = 1, 5%).

The mean total surgery time was 170.3 ± 21.3 min, with a mean cardiopulmonary bypass time of 143.4 ± 21.5 min and a mean aortic cross-clamp time of 80.4 ± 7.9 min. Postoperatively, patients had a brief intensive care unit stay (N = 20, 1.0 ± 0.2 days) and a mean hospital length of stay of 8.0 ± 1.9 days. No major perioperative complications were observed, including re-exploration for bleeding, stroke, or infection. Thirty-day mortality was 0%, reflecting excellent early outcomes in this minimally invasive mitral valve surgery cohort. Additional details on perioperative procedures and early postoperative outcomes are presented in Table 3.

## 4. Discussion

In this study, μMVR with the VirtuoSEW^®^ automated annular suturing system resulted in complete restoration of mitral valve function in all patients. There was marked improvement in echocardiographic measures of valve competence with no residual regurgitation or regurgitant jets observed. There were no cases of thrombus formation, ring detachment or dehiscence, or other device-related complications during early follow-up. There were no cases of perioperative myocardial infarction, stroke, bleeding, pneumonia, AF, or major infection. To the authors’ knowledge, this represents the first clinical study exploring the outcomes of μMVR using the VirtuoSEW^®^ system, demonstrating both its safety and efficacy.

The VirtuoSEW^®^ device [5] is specifically designed to optimize and standardize mitral annuloplasty suturing. By automating mattress suture placement, the device allows the surgeon to advance a curved needle through the annulus with a single lever squeeze, delivering consistent bite width and depth that may improve reproducibility compared with conventional hand-sewn techniques. The articulating shaft and jaws provide enhanced access to the annulus, even in minimally invasive approaches. The retractable needle design eliminates exposed sharps during handling, offering both ergonomic and safety advantages. In our experience, placement of annuloplasty sutures with VirtuoSEW^®^ appeared to be faster and more consistent than manual suturing; however, comparative studies need to be done. Other published studies of related automated devices in minimally invasive valve surgery have also demonstrated favorable cardiopulmonary bypass and cross-clamp times, suggesting that automation of the most time-consuming step of ring implantation may further reduce ischemic duration [10,11,12,13]. The device’s capacity for consistent, precise suture placement, combined with its safety features, facilitated a smooth and uniform annular suture line in our cohort. Although granular operative time data were not collected in this small series, the reproducibility and ergonomic design of VirtuoSEW^®^ suggest a clear potential for improving procedural efficiency, particularly in minimally invasive or complex mitral repairs [5].

The VirtuoSEW^®^-assisted annuloplasty combines the durability of conventional sutured repair with the efficiency of an automated device. Compared to conventional hand-sewn repair, this automated device could reduce the steep learning curve of endoscopic minimally invasive surgery by standardizing annular suture deployment. Although our study was not comparative, the findings suggest that VirtuoSEW^®^ may help extend the benefits of mitral valve repair (preserved ventricular function, avoidance of prosthesis) into even more complex or reoperative cases, without sacrificing speed or safety.

Evolving technology for automated suturing and fastening in µMVr, such as the Mi-STITCH^®^, Mi-KNOT^®^, BOBsnare^TM^ and VirtuoSEW^®^, can be complementary, further facilitating precise annuloplasty suture placement and chordal attachment in less invasive mitral valve repair surgery [5,14,15,16,17,18,19]. By standardizing bite width and depth while incorporating ergonomic and safety features, these devices can lower dexterity demands, reduce variability, and shorten cross-clamp and bypass times.

This preliminary study has several limitations. It is a single-center, non-randomized series of modest size, without a control group for direct comparison to manual suturing. We would like to clarify that the relatively limited number of cases using the VirtuoSEW^®^ system—20 procedures over the 3-year study period—reflects the constrained availability of the devices during this early clinical adoption phase. While our center performed a higher total volume of totally endoscopic mitral valve repairs during the same period, only a subset could be conducted with the VirtuoSEW^®^ system. With a mean follow-up of 9.4 ± 2.1 months, including echocardiography at 1 month, we cannot yet comment on the longer-term durability of repair with VirtuoSEW^®^. The cohort patients were relatively typical (age ~ 60, mostly degenerative pathology), but larger trials are needed to confirm that these outcomes hold across broader populations. Future research should prospectively compare VirtuoSEW^®^-assisted repair to conventional techniques in terms of operative times, repair durability, and clinical endpoints. The device may present challenges in more complex anatomical or intraoperative scenarios, which have yet to be systematically tested and validated. Importantly, long-term echocardiographic surveillance will be necessary to ensure that the excellent acute competence persists over time.

## 5. Conclusions

In conclusion, μMVR using the VirtuoSEW^®^ (LSI Solutions, Victor, NY 14564m, USA) automated suturing system was associated with excellent early outcomes: complete elimination of severe MR and no device-related complications in 20 patients. These results suggest that VirtuoSEW^®^ (LSI Solutions, Victor, NY 14564m, USA) can make mitral valve repair faster and more reproducible, preserving the advantages of a sutured annuloplasty in less invasive endoscopic surgery. Larger studies and longer follow-up will be essential to confirm these findings and to establish the role of this technology in routine cardiac surgery.

## Figures and Tables

**Table 1:** *Baseline characteristics, diagnoses, and comorbidities of the study population*.

Diagnosis (N, %)		**N**	**%**
	Mitral valve prolapse with severe regurgitation	10	50.0
	Degenerative mitral valve with severe regurgitation	5	25.0
	Ischemic severe mitral valve regurgitation	3	15.0
	Morbus Barlow with severe regurgitation	2	10.0
Sex (N, %)	Male	9	45.0
	Female	11	55.0
Age (median, 25th, 75th percentile)	57.5 (42.25; 62.5)		
Weight (median, 25th, 75th percentile)	90.0 (74.5, 96.6)		
Height (median, 25th, 75th percentile)	180.0 (172.5, 185.2)		
BMI (median, 25th, 75th percentile)	27.3 (25.7, 27.9)		
Comorbidities (N, %)	Hypertension	10	50.0
	Diabetes mellitus	2	10.0
	Hyperlipidemia	3	15.0
	Chronic kidney disease	2	10.0
	COPD	1	5.0
	PAD	2	10.0
	Smoker	5	25.0

At baseline, N = 10 (50%) patients are in sinus rhythm, whereas N = 10 (50%) presents with atrial fibrillation.

**Table 2:** Pre-, postoperative and follow-up echocardiographic parameters following mitral valve repair using the VirtuoSEW^®^ (LSI Solutions, Victor, NY 14564m, USA) automated annular suturing system.

Echocardiographic assessment	**Parameters**	**Preoperative**	**Postoperative**	**Follow-Up**	***p*-Value**
	Ejection fraction (mean ± SD)	52.5 ± 10.9	51.5 ± 11.2	53.0 ± 8.1	0.471
	LVIDd (mean ± SD)	58.1 ± 5.9	52.7 ± 5.9	52.0 ± 2.4	<0.001
	LVIDs (mean ± SD)	37.2 ± 9.0	35.7 ± 6.9	35.7 ± 10.9	<0.001
	IVSd (mean ± SD)	12.5 ± 2.0	12.4 ± 1.8	11.5 ± 1.0	0.230
	LVPWTd (mean ± SD)	10.8 ± 1.5	10.4 ± 1.5	10.5 ± 0.6	0.230
	LA (mean ± SD)	58.0 ± 9.6	52.1 ± 7.2	52.7 ± 7.5	0.019
	TAPSE (mean ± SD)	23.6 ± 3.8	23.8 ± 5.2	24.2 ± 4.3	0.275
	AV diameter (mean ± SD)	26.1 ± 3.1	26.1 ± 3.1	26.1 ± 3.1	N/A
	AV velocity (mean ± SD)	1.4 ± 0.2	1.4 ± 0.2	1.4 ± 0.2	N/A
	AV mean PG (mean ± SD)	7.5 ± 3.6	7.4 ± 3.7	7.4 ± 3.7	N/A
	Severe mitral regurgitation (N, %)	20 (100)	0 (0)	0 (0)	N/A
	Mitral valve annulus diameter (mean ± SD)	42.0 ± 5.3	34.2 ± 2.2	34.2 ± 2.1	0.001
	Vena contracta (mean ± SD)	8.4 ± 1.3	2.0 ± 1.6	2.0 ± 1.6	<0.001
	Regurgitant volume (mean ± SD)	73.5 ± 9.3	5.5 ± 5.3	5.5 ± 5.3	0.019
	Severe tricuspid regurgitation (N, %)	3 (15.0)	2 (15.0)	0 (0)	N/A
	Right ventricular systolic pressure (median, 25th, 75th percentile)	37.0 (29.0; 56.7)	36.5 (25.7; 53.7)	30.1 (24.2; 45.6)	0.109

**Table 3:** Perioperative surgical procedures and early postoperative outcomes in the study cohort.

Mitral Valve Repair Technique N (%)		N	%
Annuloplasty ring used	Physio-ring	12	60
	Memo 3D ring	4	20
	Memo 4D ring	4	20
Leaflet repair technique	Leaflet plication/tri-quadrangular resection	5	25
	Neochordae implantation	7	35
	Sliding plasty	2	10
	Commissural repair	1	5
	Hemibutterfly repair	1	5
Concomitant procedures	Tricuspid valve repair	1	5
	Atrial septal defect closure	1	5
**Operative data**			
Surgery time in minutes (mean ± SD)	170.3 ± 21.3		
CPB in minutes (mean ± SD)	143.4 ± 21.5		
XClamp in minutes (mean ± SD)	80.4 ± 7.9		
**Postoperative course**			
ICU stay in days (mean ± SD)	1.0 ± 0.2		
Hospital stay in days (mean ± SD)	8.0 ± 1.9		
**Perioperative complications**	Bleeding requiring re-exploration	0	0
	Stroke	0	0
	Infections	0	0
	30-day mortality	0	0

## Data Availability

The data presented in this study are available upon request from the corresponding author due to legal and anonymity restrictions.
